# Seroprevalence of SARS-CoV-2 antibodies among Japanese healthcare workers from 2020 to 2022 as assayed by two commercial kits

**DOI:** 10.1038/s41598-024-53656-2

**Published:** 2024-02-07

**Authors:** Yan Yan, Kaori Saito, Toshio Naito, Kanami Ito, Shuko Nojiri, Yuki Horiuchi, Gautam A. Deshpande, Hirohide Yokokawa, Yoko Tabe

**Affiliations:** 1 Department of General Medicine, Faculty of Medicine, Juntendo University, Hongo 2-1-1, Bunkyo-ku, Tokyo, 113-8421 Japan; 2 Department of Clinical Laboratory Medicine, Faculty of Medicine, Juntendo University, Tokyo, Japan; 3 Department of Safety and Health Promotion, Juntendo University, Tokyo, Japan; 4 Medical Technology Innovation Center, Juntendo University, Tokyo, Japan

**Keywords:** Epidemiology, Epidemiology

## Abstract

Antibody tests are used as surveillance tools for informing health policy making. However, results may vary by type of antibody assay and timing of sample collection following infection. Long-term longitudinal cohort studies on antibody assay seropositivity have remained limited, especially among Asian populations. Using blood samples obtained at health physicals (2020–2022) of healthcare workers (mass vaccinated with mRNA COVID-19 vaccines) at a Japanese medical center, we measured N-specific antibodies using two commercially available systems. Roche Elecsys Anti-SARS-CoV-2 measures total antibodies and Abbott Alinity SARS-CoV-2 IgG measures only IgG. Among 2538 participants, seroprevalence was found to be 16.6% via total antibody assay versus 12.9% by IgG-only (including grayzone) by mid-June 2022. For 219 cases with a previous PCR-confirmed infection, positivity was 97.3% using total antibody assay versus 76.3% using IgG-only assay at the 2022 health physical. Using PCR positive test date as day 0, while the positivity of the total antibody assay was retained for the entire study period (until more than 24-months post-infection), the IgG-only assay’s positivity declined after month 4. The Mantel–Haenszel test found a significant difference in the two assays’ seropositivity, between stratified groups of “within 3 months” and “4 months or more” from infection (*P* < 0.001). Our study found significant differences in seropositivity over time of total antibody versus IgG-only assays, suggesting an optimal assay for retaining sensitivity over the entire infection period when designing seroprevalence studies.

## Introduction

Severe acute respiratory syndrome coronavirus 2 (SARS-CoV-2) transmission has been reported in Japan since early 2020, with approximately 27.0 million cumulative COVID-19 cases reported as of mid-December 2022^1^. Due to more involvement of the upper respiratory tract, and possibly also due to Japan’s high vaccination coverage, infections by Omicron and its subvariants have resulted in cases with mild or no symptoms compared to previous variants^2–4^. Routine public health reporting may underestimate true numbers of infections because mild or asymptomatic cases may not be tested and are likely to remain unidentified.

By mid-November 2022, Japan’s infection rate reached 19.0% (23.3 million cumulative cases out of 122.8 million in the national population, including those with multiple infections)^5^. In contrast, a recent national N-antibody seroprevalence study in Japan (n = 8260) of donated blood revealed a national infection rate of approximately 26.5% by mid-November 2022^6^. Despite comprehensive testing and reporting systems existing nationwide in Japan, the substantially higher N-seropositive rate compared to the PCR-positive based standard public health calculations highlights the challenges in achieving accurate understanding of the infection situation.

Of the three major methods for detecting SARS-CoV-2 infection—PCR, rapid antigen tests, and antibody tests—antibody tests remain the least utilized choice. PCR tests are recommended by the WHO for confirming diagnosis in symptomatic individuals, and rapid antigen tests have the advantage of being faster for those most likely at risk of transmitting the virus. Nonetheless, antibody tests, which detect the host response to infection or vaccination, have proven to be useful surveillance tools to inform public policy^7^.

The nucleocapsid protein (NCP) of SARS-CoV-2 is essential for viral genome condensation and packaging and is quantifiably the most abundant viral protein in infected cells^8^. Currently available mRNA COVID-19 vaccines do not contain NCP or nucleotides encoding NCP. As such, anti-SARS-CoV-2-nucleocapsid (N) antibodies can theoretically be used to identify individuals who have been previously infected with SARS-CoV-2. For this reason, it may be helpful to monitor N-specific antibody levels to determine seroprevalence in targeted populations, especially in those with high rates of vaccination with an mRNA vaccine^9^.

Previous studies have reported that antibody seropositivity (magnitude and detectability) is driven by disease severity, as well as both timing and type of assay^10–13^. Although comparison studies on the clinical performance of various SARS-CoV-2 antibody assays—total antibody, IgG, and IgM—have been previously published, most of these studies utilized samples from recently infected persons, typically within 6 months from COVID-19 symptom onset or PCR positive dates^12,14–18^. Little is known about test performance regarding duration of seropositivity of the commonly used antibody tests—an important feature to determine choice of test in future seroprevalence studies.

We previously reported that due to our strict infection control protocol and robust vaccine campaigns (mostly with Pfizer/BioNTech BNT162b2 and Moderna mRNA-1273), the seroprevalence among the HCWs of Juntendo University Hospital remained extremely low (0.3% by mid-July 2020; 1.6% by mid-June 2021). In the other words, 98.4% of our HCWs were infection-naïve by mid-2021^19–22^. We also reported that by mid-June 2022, the seroprevalence rate increased to 16.7%, mainly due to spread of the Omicron variant^23^. In these studies, data was obtained from annual occupational health physical samples and all were tested using a total antibody assay (Roche Elecsys Anti-SARS-CoV-2).

In order to demonstrate and clarify the variation in seropositivity reporting in epidemiological surveys, in the current study we tested all samples from the 2022 health physical using two assays: the total antibody assay noted above (Roche Elecsys Anti-SARS-CoV-2), as well as an IgG-only assay (Abbott Alinity SARS-CoV-2 IgG). Additionally, for participants with PCR-confirmed infections, we tested blood samples from their 2020 and 2021 annual health physicals and examined the seropositivity over time, using the PCR positive date as day 0.

## Results

A total of 2538 HCWs gave consent for use of three consecutive years (2020 through 2022) of data obtained from blood samples. Characteristics of the studied population are shown in Table 1. 64.3% were women and 92.0% were aged 20–59 years old (mean = 38.0; SD, ± 12.0). 609 (24.0%) had close contacts with COVID-19 patients as frontline healthcare providers. As of mid-June 2022, 97.5% had received 2 or more doses of a COVID-19 mRNA vaccine.

**Table 1:** N-specific antibody testing results by serology assay type among HCW participants at the 2022 health physical (n = 2538)^a^.

	Total		Roche Elecsys Anti-SARS-CoV-2		Abbott Alinity SARS-CoV-2 IgG			
	n	%	Negative n (%)	Positive n (%)	Negative n (%)	Grayzone n (%)	Positive n (%)	Grayzone/Positive n (%)
Overall	2538	(100.0)	2118 (83.5)	420 (16.6)	2211 (87.1)	136 (5.4)	191 (7.5)	327 (12.9)
Age								
20–29	597	(23.5)	462 (77.4)	135 (22.6)	494 (82.7)	37 (6.2)	66 (11.1)	103 (17.3)
30–39	747	(29.4)	615 (82.3)	132 (17.7)	640 (85.7)	47 (6.3)	60 (8.0)	107 (14.3)
40–49	638	(25.1)	532 (83.4)	106 (16.6)	555 (87.0)	38 (6.0)	45 (7.1)	83 (13.0)
50–59	355	(14.0)	316 (89.0)	39 (11.0)	329 (92.7)	10 (2.8)	16 (4.5)	26 (7.3)
60–69	164	(6.5)	158 (96.3)	6 (3.7)	257 (95.7)	4 (2.4)	3 (1.8)	7 (4.3)
70 or older	37	(1.5)	35 (94.6)	2 (5.4)	36 (97.3)	0 (0.0)	1 (2.7)	1 (2.7)
Sex								
Male	907	(35.7)	754 (83.1)	153 (16.9)	788 (86.9)	47 (5.2)	72 (7.9)	119 (13.1)
Female	1631	(64.3)	1364 (83.6)	267 (16.4)	1423 (87.2)	89 (5.5)	119 (7.3)	208 (12.8)
Profession								
Doctor	748	(29.5)	612 (81.8)	136 (18.2)	638 (85.3)	46 (6.1)	64 (8.6)	110 (14.7)
Nurse	820	(32.3)	650 (79.3)	170 (20.7)	696 (84.9)	51 (6.2)	73 (8.9)	124 (15.1)
Paramedical staff^b^	399	(15.7)	360 (90.2)	39 (9.8)	366 (91.7)	13 (3.3)	20 (5.0)	33 (8.3)
Administration staff	391	(15.4)	336 (85.9)	55 (14.1)	349 (89.3)	17 (4.3)	25 (6.4)	42 (10.7)
Other	180	(7.1)	160 (88.9)	20 (11.1)	162 (90.0)	9 (5.0)	9 (5.0)	18 (10.0)
Close contacts with COVID-19 patients								
No	1929	(76.0)	1630 (84.5)	299 (15.5)	1698 (88.0)	106 (5.5)	125 (6.5)	231 (12.0)
Yes	609	(24.0)	488 (80.1)	121 (19.9)	513 (84.2)	30 (4.9)	66 (10.8)	96 (15.8)
No. of vaccine doses received (mRNA vaccines)								
0 dose	54	(2.1)	45 (83.3)	9 (16.7)	47 (87.0)	2 (3.7)	5 (9.3)	7 (13.0)
1 dose	9	(0.4)	7 (77.8)	2 (22.2)	7 (77.8)	0 (0.0)	2 (22.2)	2 (22.2)
2 doses	144	(5.7)	110 (76.4)	34 (23.6)	113 (78.5)	14 (9.7)	17 (11.8)	31 (21.5)
3 doses	2115	(83.3)	1749 (82.7)	366 (17.3)	1837 (86.9)	115 (5.4)	163 (7.7)	278 (13.1)
4 doses	216	(8.5)	207 (95.8)	9 (4.2)	207 (87.1)	136 (5.4)	191 (7.5)	327 (12.9)
PCR-confirmed COVID-19 infection?								
Yes	219	(8.6)	6 (2.7)	213 (97.3)	52 (23.7)	59 (26.9)	108 (49.3)	167 (76.3)
No	2319	(91.4)	2112 (91.1)	207 (8.9)	2159 (93.1)	77 (3.3)	86 (3.6)	160 (6.9)

^a^ Data in this table reflect the situation by the timepoint of 2022 annual health physical.

^b^ Paramedical staff includes clinical laboratory personnel, pharmacists, rehabilitation specialists, etc.

Among all 2538 participants, 16.6% (420/2538; 95% confidence interval [CI] 15.0–18.2) were found to be N-seropositive by total antibody assay by mid-2022. Using IgG-only assay, prevalence was 12.9% (327/2538; 95% CI 11.5–14.4), including 191 positive and 136 grayzone results (In this study, grayzone results of the IgG-only assay are considered positive) **(**Table 1**)**. Multivariate logistic regression analysis showed that compared to participating HCWs aged 60 or above, being aged 59 or younger is significantly associated with higher seropositivity, tested by the total antibody assay (OR = 4.96; 95% CI 2.41–10.21; *p* = 0.000), and also by the IgG-only assay (OR = 3.56; 95% CI 1.72–7.36; *p* = 0.001) (Table 2). Having 2 doses of vaccine or less was significantly higher with seropositivity when tested by the IgG-only assay (*p* = 0.006), but didn’t prove to be a significant factor when tested by the total antibody assay (*p* = 0.058) (Table 2).

**Table 2:** Multivariate logistic regression analysis for seropositivity among participating HCWs at the 2022 health physical, by serology assay type (n = 2538).

Parameter	Participants in total (n)	Roche Elecsys Anti-SARS-CoV-2				*p*-value	Abbott Alinity SARS-CoV-2 IgG				*p*-value
		Positive Results (n)	Positive (%)	OR	[95% CI]		Positive Results^a^ (n)	Positive (%)	OR	[95% CI]	
Total	2538	420	16.5				327	12.9			
Age											
≥ 60 (reference)	201	8	4.0				8	4.0			
≤ 59	2337	412	17.6	4.96	[2.41–10.21]	0.000	319	13.6	3.56	[1.72–7.36]	0.001
Sex											
Male (reference)	907	153	16.9				119	13.1			
Female	1631	267	16.4	0.90	[0.72–1.12]	0.342	208	12.8	0.92	[0.72–1.17]	0.481
Close contacts with COVID-19 patients											
No (reference)	1929	299	15.5				231	12.0			
Yes	609	121	19.9	1.23	[0.97–1.56]	0.085	96	15.8	1.28	[0.99–1.67]	0.063
No. of vaccine doses											
3 or more (reference)	2331	375	16.1				287	12.3			
≤ 2	207	45	21.7	1.40	[0.99–1.99]	0.058	40	19.3	1.67	[1.15–2.41]	0.006

^a^Positive results include those of grayzone for Abbott Alinity SARS-CoV-2 IgG assay.

There were 219 participants with recorded PCR-confirmed infection of the past 3 years and 2319 without. Among the 219 PCR-confirmed cases, by using sera from the 2022 health physical, total antibody assay detected 213 seropositive while 6 were seronegative; IgG-only assay found 167 seropositive (including 59 grayzone), and 52 seronegative. Among the 2319 participants without record of a PCR-confirmed infection, the total antibody assay detected 207 positive cases, accounting for 49.3% of all 420 seropositive cases identified with the total antibody assay at the 2022 health physical. For IgG-only assay, it detected a total of 160 positive or grayzone cases, accounting for 48.9% of all identified 327 seropositive or grayzone cases at the 2022 health physical (Table 1).

**Figure 1:**
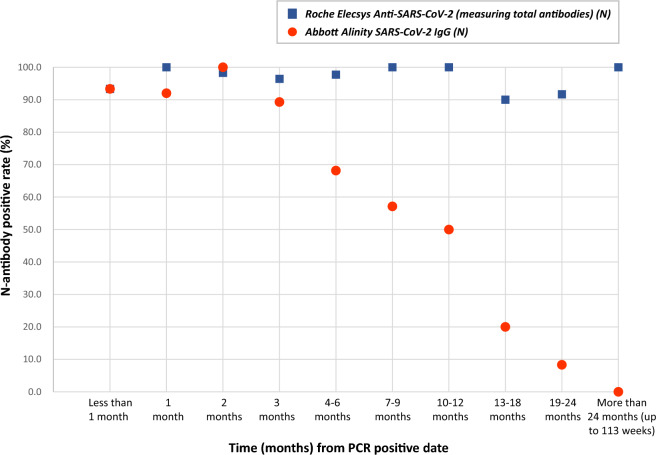
Seropositivity among participants with a PCR-confirmed infection at the 2022 health physical, by serology assay type (n = 219). For Abbott Alinity SARS-CoV-2 IgG assay, positive results included those in grayzone. PCR positive dates were reported by HCW participants with PCR tests done either at JUH or other medical facilities. “Months” in the figure means “complete months” between PCR positive date and blood sample collection date.

Distribution of positive and negative results among the overall 2538 participants by type of assay is shown in Table 3. At the 2022 heath physical, 117 samples were positive with total antibody assay but negative with IgG-only assay; another 24 samples were positive (including grayzone) with IgG-only assay but negative with total antibody assay. Assay concordance was substantial (k, 0.78; 95% CI 0.75–0.82). However, the results of our developed logistic model for estimating the risk of infection showed low accuracy for both assays. The scores of c-statistics were 0.57 (95% CI 0.53–0.60) for the total Ig assay, and 0.57 (95% CI, 0.54 to 0.60) for the IgG-only assay (S Fig. 1a,b).

**Table 3:** Distribution of positive and negative results at the 2022 health physical, by serology assay type (n = 2538)^a^.

Roche Elecsys Anti-SARS-CoV-2 results (n)	Abbott Alinity SARS-CoV-2 IgG results (n)			Percentage agreement	*kª* Statistic (95% CI)
	Positive results^b^	Negative results	Total		
Positive	303	117	420	94.4%	0.78 (0.75–0.82)
Negative	24	2094	2118		
Total	327	2211	2538		

^a^Data in this table reflect the testing results by the timepoint of 2022 annual health physical.

^b^Positive results include those of grayzone for Abbott Alinity SARS-CoV-2 IgG assay.

Among the participating HCWs with a PCR-confirmed infection history, positivity with respect to months from PCR positive date by assay types is shown in Table 4, and visually presented in Fig. 1. The Mantel–Haenszel test found a significant difference in the two assays’ seropositivity, between stratified groups of “within 3 months” and “4 months or more” from infection (using PCR positive date as day 0) (*P* < 0.001). Detailed weekly positivity by assay types is shown in S Table 1. While the total antibody remained positive throughout the study period (more than 24 months), IgG-only assay’s positivity began to decline over time, starting from month 4. Complete testing results by serology type, categorized by year, for each of the 219 participant HCWs with a PCR-confirmed infection are shown in S Table 2.

**Table 4:** Seropositivity among participants with a PCR-confirmed infection at the 2022 health physical, by serology assay type (n = 219).

No. of months^a^ from the positive PCR test	Total	Roche Elecsys Anti-SARS-CoV-2			Abbott Alinity SARS-CoV-2 IgG			Grayzone/Positive	% Grayzone/Positive	Mantel–Haenszel *p*-value
		Negative	Positive	% Positive	Negative	Grayzone	Positive			
< 1 month	15	1	14	93.3	1	2	12	14	93.3	< 0.001
1 month	25	0	25	100.0	2	2	21	23	92.0	
2 months	58	1	57	98.3	0	13	45	58	100.0	
3 months	28	1	27	96.4	3	14	11	25	89.3	
** ≤ 3 months**	126	3	123	97.6	6	31	89	120	95.2	
4–6 months	44	1	43	97.7	14	20	10	30	68.2	
7–9 months	14	0	14	100.0	6	5	3	8	57.1	
10–12 month	12	0	12	100.0	6	1	5	6	50.0	
13–18 months	10	1	9	90.0	8	1	1	2	20.0	
19–24 months	12	1	11	91.7	11	1	0	1	8.3	
> 24 months	1	0	1	100.0	1	0	0	0	0.0	
** ≥ 4 months**	93	3	90	96.8	46	28	19	47	50.5	
** Total**	219	6	213	97.3	52	59	108	167	76.3	

^a^Months” in the above table means “complete months” between PCR positive date and blood sample collection date.

## Discussion

Comparing the two commercially available COVID-19 antibody assays, our study revealed a difference regarding seropositive rate—16.6% in the total antibody assay versus 12.9% in the IgG—only assay at the 2022 health physical. Our findings are corroborated by previous studies in Japan, which have also reported that N-specific serological results varied by assay type. In June 2020, a population-based seroprevalence surveillance study by the National Institute of Infectious Disease (n = 1971 in Tokyo; n = 2970 in Osaka; n = 3009 in Miyagi prefecture) found inconsistent results between the two assays^24^.

Regarding seropositivity over time by assay type, notably, we found that while the total antibody assay generally retained 100% seropositivity from PCR-confirmed infection date through the entire study period, seropositivity of the IgG-only assay dropped beginning around month 4. In a review study (including 178 studies with 527 test evaluations), Fox et al. reported an average 94.3% sensitivity for total antibodies and 89.8% for IgG-only assays during the convalescent phase of infection (up to 100 days after onset of symptoms)^14^. In a study with a longer study period of up to 33 weeks of 5788 Irish HCWs, Allen et al. reported that positivity with an IgG-only assay began to decline at 21 weeks after confirmed infection^12^. Another study evaluating the cross-reactivity of N-specific antibodies in COVID-19 patients by ELISA using recombinant N protein from the SARS-CoV-2 mutant strain showed a marked decrease in N-specific antibodies 1 year after infection, with N-IgG antibodies detected in only about 17% of patients at 14 months after PCR-confirmed infection^25^.

Our findings from this longitudinal study were consistent with previous studies—the seropositivity of IgG-only assays decline over time from infection^12,25^. Yet, compared to the Irish study, we found that IgG-only seropositivity started to decline at even an earlier time (month 4). As Asian ethnicity has been reported to be a factor associated with IgG seronegativity, and 100% of our study population was Asian, this may be an associated factor for faster decline of IgG seropositivity in our study. The magnitude of effect that regional or ethnic differences exert on the early decline of SARS-CoV-2 N IgG antibodies warrants further investigation. In contrast, the total antibody assay in our study, which measures N-Ta, showed little decrease in positivity over time, suggesting that differences in the isotype of anti-N antibody detected may determine the availability of N antibody long after infection. Serological assays for antibodies to SARS-CoV-2 often use different epitopes for the same protein; this may therefore affect detection rates^26^. In addition, these assays are not cross-calibrated, making direct comparisons between the various assays difficult.

Although our findings suggested that total antibody assay would be a more appropriate choice for COVID-19 epidemiological studies, our developed logistic model for estimating the risk of infection showed low accuracy for both assays (scores of c-statistics as 0.57 for the total Ig assay; 0.57 for the IgG-only assay). Further investigation is warranted to examine the seropositivity’s change over time by assay type.

For the IgG-only assay in particular, while its positivity among those with a PCR-confirmed infection (n = 219) was 76.3% through the entire study period, our study found 59 in the grayzone versus 108 with clearly positive results (Table 4). Excluding grayzone results in further sub-analysis, IgG-only assay positivity dropped to 49.3%, substantially lower than the 97.3% seropositivity in the total antibody assay. Our findings suggest the importance of including grayzone results when using this IgG-only assay.

While 219 (out of 2538) reported a previously PCR-confirmed infection in this study, we identified 420 seropositive cases with the total antibody assay and 327 seropositive cases with the IgG-only assay in the year 2022. Close to 50% (49.3% [207 out of 420] using total antibody assay; 48.9% [160 out of 327] using IgG-only assay) of the seropositive cases were found among persons without awareness of COVID-19 infection. Considering this hospital’s robust vaccination campaigns and strict infection-control measures including daily temperature checks and PCR tests for close contacts of confirmed cases, symptomatic cases should have been detected thoroughly. Our findings indicate that these asymptomatic infection cases wouldn’t have been identified if PCR positive tests were utilized as the only identification tool. Importantly, our findings support that antibody testing, including both total antibody assay and IgG-only assay, remains a useful tool to monitor seroprevalence of COVID-19 among target populations, especially those with a high vaccination rate and/or infection histories who would display mild or no symptoms when infected.

### Limitations

This study has several limitations worth addressing. First, CT values of PCR tests and data regarding symptoms were not available for all previously infected participants, precluding speculation on possible associations between assay performance and disease severity. Second, although reinfection is considered limited among this study population, undetected reinfection may exist, which may shorten the interval between infection and serological testing, therefore affecting seropositivity of antibody assays over time. Third, since there were a number of infection cases without awareness, the reported PCR positive cases didn’t represent all actual infection cases, which makes analyzing sensitivity and specificity of the two commercially available assays unavailable. Fourth, the majority of participants in our sample were younger than 60 years old and included a large group of young female nurses, not a fully representative sample of the population of the Tokyo metropolitan area. Additionally, although this medical center has strict infection control protocols, the participating HCWs (including close contacts with COVID-19 patients) had a higher risk to be exposed to infected patients and therefore a higher risk for COVID-19 infection, which may not reflect the real-world practices of the population in Tokyo. As such, our findings should be interpreted with caution.

### Conclusion

In countries with high vaccination coverage and/or high percentage of natural COVID-19 infection, many cases remain asymptomatic, potentially causing underestimation of SARS-CoV-2 prevalence. Though our study shows that antibody testing remains a useful tool to monitor seroprevalence of COVID-19 among target populations, especially among those with possible infection without awareness, we highlight the fact that seropositivity varies significantly by assay type and from time of infection. Although further investigation is warranted, in this 3-year longitudinal study, we found that IgG-only assay’s positivity declines over time, indicating that the total antibody assay would be a more appropriate choice for COVID-19 epidemiological studies. In conclusion, our findings suggest an optimal assay for retaining sensitivity over the entire infection period when designing seroprevalence studies.

## Methods and materials

### Study design

This is a comparative study using two commercially available nucleocapsid protein (N-specific) SARS-CoV-2 antibody assays—a total antibody assay (Elecsys Anti-SARS-CoV-2, Roche Diagnosis, Basel, Switzerland) versus an IgG-only assay (Alinity SARS-CoV-2 IgG, Abbott Laboratories, Chicago, IL, USA)—to compare seropositivity over time from infection. Study participants were 2538 HCWs of Tokyo-based Juntendo University Hospital, who gave consent for using blood samples from annual occupational health physicals between 2020 and 2022. Information on participants’ COVID-19 vaccination records (receiving either Pfizer/BioNTech BNT162b2 or Moderna mRNA-1273 vaccines) and their demographic information were extracted from JUH employee charts. Previous PCR-confirmed infection dates were self-reported and recorded in the hospital’s electronic system. Detailed information regarding the hospital’s baseline infection control measures and vaccination is described in Supplementary Information.

Reinfection is an important factor to consider in longitudinal serological studies, with undetected reinfection effectively shortening the interval between infection and serological testing, and therefore affecting seropositivity of antibody assays over time^16,18^. However, reinfection is considered to be limited within the study period (July 2020 to June 2022), due to the hospital’s strict infection control protocol and robust vaccine campaigns^19–21,27–29^ (Supplementary Information).

This study was approved by Juntendo Ethical Committee (IRB # M20-0089-M01) and was performed in accordance with the Helsinki declaration.

### Serological testing

The total antibody assay was used to test sera at each year’s health physicals from 2020 through 2022 for all participating healthcare workers. N-positivity results identified in this total antibody assay for those 3 years have been published^19,20,23^. In this study, the IgG-only assay was used to test all 2538 samples at the 2022 health physical. In addition, for those with a PCR-confirmed infection, samples of 2020 to 2022 were tested by the IgG-only assay as well.

Antibodies against the N-specific SARS-CoV-2 were measured for total antibody assay on Cobas e 801 analyzer (Roche Diagnosis, Basil, Switzerland) and for IgG-only assay on the Alinity platform (Abbott Laboratories, Chicago, IL, USA) according to manufacturer instructions. The total antibody assay detects N-specific total immunoglobulins by electrochemiluminescence immunoassay and results are presented qualitatively in the form of a cut-off index (COI; signal sample/cut-off), with COI ≥ 1.0 being interpreted as positive^30–32^. The IgG-only assay is a chemiluminenscent microparticle immunoassay that detects IgG antibodies to the N-protein of SARS-CoV-2. The assay threshold of ≥ 1.4 (sample to calibrators [S/C]) were interpreted as positive^33,34^. In October 2020, manufacturer guidance on the IgG-only assay was updated to include an optional editable “grayzone” with a S/C index range of 0.5–1.39 (Abbott Diagnostics Product Information Letter PI1060-2020)^35–37^. The interpretation of S/C indices used in this study was as follows: negative, < 0.5; grayzone, 0.5 to < 1.4; and positive, ≥ 1.4. The grayzone results as presumptive positives are likely to be positive on other testing platforms and may add value to individual serological tests by indicating the need for additional testing^12^.

Antibodies targeting the nucleocapsid (N) protein of SARS-CoV-2 were reportedly not detected in samples collected during the pre-pandemic period (2015–2019)^38^. However, to confirm that the N antibody positivity is due to SARS-CoV-2 infection and not to pre-existing antibodies induced by other coronaviruses, it is advisable to perform the assay using sera from the pre-pandemic period, which could not be done in this study.

### Statistical analysis

Assay concordance was accessed using Cohen’s kappa statistic for difference in proportions. Cohen’s kappa coefficient (k) measures the level of agreement between assays, taking into account the possibility of the agreement occurring by chance. The k statistic varies from 0 to 1, where 0 indicates agreement equivalent to chance, and 0.00 to 0.20, 0.21 to 0.40, 0.41 to 0.60, 061 to 0.80, 0.81 to 0.99 representing slight, fair, moderate, substantial, and almost perfect agreement, respectively. A kappa of 1 indicates perfect agreement^39,40^.

The seroprevalence is presented as crude percentages with 95% CIs. Multivariate logistic regression analysis was performed to compute ORs of seroprevalence with respect to basic characteristics. The Mantel–Haenszel test was performed to examine the two assays’ seropositivity between the stratified groups of “within 3 months” and “4 months or more” from infection (using PCR positive date as day 0), among the participating HCWs with a PCR-confirmed infection history. In addition, a logistic model was developed to estimate the risk of infection among participating HCWs for both assays, with variables of sex, age, having close contacts to COVID-19 patients, and the number of vaccine doses. Receiver operating characteristic (ROC) curves were used to estimate model discrimination by the c-statistics or area under the curve (AUC). IBM SPSS Statistics 29 and R version 4.3.1 were used. A two-tailed *p* < 0.05 was considered significant.

### Ethics declarations

This study was approved by the Institutional Review Board (IRB) at Juntendo University Hospital, Japan (IRB # M20-0089-M01). Informed consent was given by all participating health care workers.

### Supplementary Information

Supplementary Information.

## Data Availability

All data generated or analyzed during this study are included in this article.
