# Supplementary material for: Seroprevalence of SARS-CoV-2 antibodies among Japanese healthcare workers from 2020 to 2022 as assayed by two commercial kits
Source: Sci Rep. 2024 Feb 7;14:3102. doi: 10.1038/s41598-024-53656-2 (PMC10850062; doi:10.1038/s41598-024-53656-2)
Supplement: Supplementary file 1 — Supplementary Information. [file 41598_2024_53656_MOESM1_ESM.docx]

**Supplementary Information**

**Article Title:** Seroprevalence of SARS-CoV-2 antibodies among Japanese healthcare workers from 2020 to 2022 as assayed by two commercial kits

**Authors:** Yan Yan, Kaori Saito, Toshio Naito, Kanami Ito, Shuko Nojiri, Yuki Horiuchi, Gautam A. Deshpande, Hirohide Yokokawa, Yoko Tabe

1. Description of the hospital’s baseline infection control measures and vaccination

2. Explanation regarding limited reinfection during the study period

3. S Figure 1a. Receiver operating characteristic (ROC) curve for the Roche Elecsys Anti-SARS-CoV-2 assay (n=2,538)

S Figure 1b. Receiver operating characteristic (ROC) curve for the Abbott Alinity SARS-CoV-2 IgG assay (n=2,538)

4. S Table 1. Seropositivity among participants with a PCR-confirmed infection by the 2022 health physical, by serology assay type, weekly (n=219)

5. S Table 2. N-Specific antibody testing results among participants with a PCR-confirmed infection, by serology assay type by year (n=219)

**1. Description of the hospital’s baseline infection control measures and vaccination**

***Infection control measures***

Juntendo University Hospital (JUH) is a 1,051-bed academic medical center in Japan. Since the outbreak of the COVID-19 pandemic, strict infection control measures have been implemented. At the hospital, masks are universally required for all HCWs and patients in all facilities including outpatient clinics and wards (when patients’ conditions allow). For HCWs, in addition to universal masking, face shield or eye protection is required when encountering patients; additionally, use of N95 respirators when caring for patients with suspected or confirmed COVID-19 is mandated. Temperature checks are performed daily at the workplace, with COVID-19 symptoms requiring further examination. Dining with more than 3 non-family members outside work hours is discouraged per hospital policy. Close contacts of confirmed cases are PCR-tested and quarantined.

***Vaccination***

On-site employee vaccinations with BNT162b2 began on March 17, 2021; the first dose from March 17 to April 23, and April 7 to May 19 for the second dose. mRNA-1273 vaccines were not available at JUH until September 1. The booster dose mass vaccination was administrated from December 13 to 24, 2021 with two mRNA vaccines (BNT162b2 and mRNA-1273). While more than 80% of the HCWs received COVID-19 vaccines with our vaccination campaign programs, some chose other locations such as the government-run mass vaccination sites with different schedules. Vaccination dates and the type of vaccines are recorded.

**2. Explanation regarding limited reinfection during the study period**

Due to our hospital’s strict infection control protocol and effective vaccine campaigns, the seroprevalence among HCWs remained extremely low (0.3% by mid-July 2020; 1.6% by mid-June 2021). In the other words, 98.4% of our HCWs were infection-naïve by mid-2021 [1–4]. Infection was considered to be concentrated after the spread of Omicron variant, which was first detected by mid-Dec. 2021 [5]. Because of the timely and effective booster vaccination campaign at the end of Dec. 2021, this studied population was highly vaccinated with a third dose booster (mRNA vaccines) when Omicron started to spread in the Tokyo Metropolitan area. Previous studies have demonstrated that mRNA vaccines were associated with high protection against SARS-CoV-2 infection within 6 months [6,7]. Thus, reinfection between early 2022 (the spread of the Omicron variant in the Tokyo area) to our serological testing in the mid-2022 is considered to be limited.

**References:**

1. Fukuda H, Seyama K, Ito K, et al. SARS-CoV-2 seroprevalence in healthcare workers at a frontline hospital in Tokyo. *Sci Rep.* **11**(1):8380. doi: 10.1038/s41598-021-87688-9 (2021).

2. Igawa G, Ai T, Yamamoto T, et al. Antibody response and seroprevalence in healthcare workers after the BNT162b2 vaccination in a University Hospital at Tokyo. *Sci Rep.* **12**(1):8707. doi: 10.1038/s41598-022-12809-x (2022).

3. Yan Y, Naito T, Tabe Y, et al. Increased delta variant SARS-CoV-2 infections in a highly vaccinated medical center in Japan. *Vaccine.* **40**(23):3103-3108. doi: 10.1016/j.vaccine.2022.04.029 (2022).

4. Okumura N, Tsuzuki S, Saito S, et al. The first eleven cases of SARS-CoV-2 Omicron variant infection in Japan: A focus on viral dynamics. [*Glob Health Med.*](https://www.ncbi.nlm.nih.gov/pmc/articles/PMC9066457/) **4**(2): 133–136. doi: [10.35772/ghm.2021.01124](https://doi.org/10.35772%2Fghm.2021.01124) (2022).

5. Tokyo COVID-19 Infection Control Center, Metropolitan Government. Detection of the Omicron variant. https://www.metro.tokyo.lg.jp/tosei/hodohappyo/press/2021/12/22/31.html. (accessed Nov 10, 2023)

6. Hall V, Foulkes S, Insalata F, et al. Protection against SARS-CoV-2 after Covid-19 Vaccination and Previous Infection. *N Engl J Med.***386**(13):1207-1220. doi: 10.1056/NEJMoa2118691 (2022).

7. Matuchansky C. Protection against SARS-CoV-2 after Vaccination and Previous Infection. *N Engl J Med*. **386**(26):2534. doi: 10.1056/NEJMc2205618 (2022).

**3. S Figure 1a. Receiver operating characteristic (ROC) curve for the Roche Elecsys Anti-SARS-CoV-2 assay (n=2,538)**

**3. S Figure 1b. Receiver operating characteristic (ROC) curve for the Abbott Alinity SARS-CoV-2 IgG assay (n=2,538)**

**4. S Table 1. Seropositivity among participants with a PCR-confirmed infection by the 2022 health physical, by serology assay type, weekly (n=219)**

| **No. of weeks from the positive PCR test** |  |  | **Roche Elecsys** **Anti-SARS-CoV-2** | | |  | **Abbott Alinity SARS-CoV-2 IgG** | | | | |
| --- | --- | --- | --- | --- | --- | --- | --- | --- | --- | --- | --- |
|  |  | **Total** | **Negative** | **Positive** | **% Positive** |  | **Negative** | **Grayzone** | **Positive** | **Grayzone/Positive** | **% Grayzone/Positive** |
| 2 |  | 2 |  | 2 | 100.0 |  |  |  | 2 | 2 | 100.0 |
| 3 |  | 11 | 1 | 10 | 90.9 |  | 1 | 2 | 8 | 10 | 90.9 |
| 4 |  | 5 |  | 5 | 100.0 |  |  |  | 5 | 5 | 100.0 |
| 5 |  | 3 |  | 3 | 100.0 |  |  |  | 3 | 3 | 100.0 |
| 6 |  | 8 |  | 8 | 100.0 |  | 1 | 1 | 6 | 7 | 87.5 |
| 7 |  | 8 |  | 8 | 100.0 |  |  | 1 | 7 | 8 | 100.0 |
| 8 |  | 3 |  | 3 | 100.0 |  | 1 |  | 2 | 2 | 66.7 |
| 9 |  | 12 |  | 12 | 100.0 |  |  | 1 | 11 | 12 | 100.0 |
| 10 |  | 18 |  | 18 | 100.0 |  |  | 5 | 13 | 18 | 100.0 |
| 11 |  | 16 | 1 | 15 | 93.8 |  |  | 2 | 14 | 16 | 100.0 |
| 12 |  | 10 |  | 10 | 100.0 |  |  | 4 | 6 | 10 | 100.0 |
| 13 |  | 6 |  | 6 | 100.0 |  |  | 2 | 4 | 6 | 100.0 |
| 14 |  | 3 |  | 3 | 100.0 |  |  | 3 |  | 3 | 100.0 |
| 15 |  | 8 |  | 8 | 100.0 |  |  | 4 | 4 | 8 | 100.0 |
| 16 |  | 11 |  | 11 | 100.0 |  | 2 | 6 | 3 | 9 | 81.8 |
| 17 |  | 13 | 1 | 12 | 92.3 |  | 5 | 3 | 5 | 8 | 61.5 |
| 18 |  | 10 |  | 10 | 100.0 |  | 4 | 3 | 3 | 6 | 60.0 |
| 19 |  | 14 |  | 14 | 100.0 |  | 3 | 9 | 2 | 11 | 78.6 |
| 20 |  | 6 |  | 6 | 100.0 |  | 2 | 3 | 1 | 4 | 66.7 |
| 21 |  | 1 | 1 |  | 0.0 |  |  | 1 |  | 1 | 100.0 |
| 22 |  | 2 |  | 2 | 100.0 |  | 1 | 1 |  | 1 | 50.0 |
| 38 |  | 4 |  | 4 | 100.0 |  | 2 | 1 | 1 | 2 | 50.0 |
| 39 |  | 1 |  | 1 | 100.0 |  | 1 |  |  | 0 | 0.0 |
| 40 |  | 3 |  | 3 | 100.0 |  | 2 |  | 1 | 1 | 33.3 |
| 41 |  | 2 |  | 2 | 100.0 |  |  | 2 |  | 2 | 100.0 |
| 42 |  | 2 |  | 2 | 100.0 |  | 1 | 1 |  | 1 | 50.0 |
| 43 |  | 4 |  | 4 | 100.0 |  |  | 2 | 2 | 4 | 100.0 |
| 44 |  | 2 |  | 2 | 100.0 |  | 1 |  | 1 | 1 | 50.0 |
| 45 |  | 4 |  | 4 | 100.0 |  | 2 |  | 2 | 2 | 50.0 |
| 46 |  | 3 |  | 3 | 100.0 |  | 3 |  |  | 0 | 0.0 |
| 49 |  | 1 |  | 1 | 100.0 |  |  |  | 1 | 1 | 100.0 |
| 57 |  | 1 |  | 1 | 100.0 |  |  | 1 |  | 1 | 100.0 |
| 58 |  | 1 |  | 1 | 100.0 |  | 1 |  |  | 0 | 0.0 |
| 70 |  | 1 |  | 1 | 100.0 |  | 1 |  |  | 0 | 0.0 |
| 72 |  | 1 |  | 1 | 100.0 |  | 1 |  |  | 0 | 0.0 |
| 74 |  | 1 | 1 |  | 0.0 |  | 1 |  |  | 0 | 0.0 |
| 75 |  | 3 |  | 3 | 100.0 |  | 3 |  |  | 0 | 0.0 |
| 76 |  | 1 |  | 1 | 100.0 |  | 1 |  |  | 0 | 0.0 |
| 80 |  | 1 |  | 1 | 100.0 |  |  |  | 1 | 1 | 100.0 |
| 85 |  | 1 |  | 1 | 100.0 |  | 1 |  |  | 0 | 0.0 |
| 88 |  | 1 |  | 1 | 100.0 |  | 1 |  |  | 0 | 0.0 |
| 90 |  | 1 |  | 1 | 100.0 |  | 1 |  |  | 0 | 0.0 |
| 94 |  | 2 |  | 2 | 100.0 |  | 2 |  |  | 0 | 0.0 |
| 97 |  | 1 |  | 1 | 100.0 |  | 1 |  |  | 0 | 0.0 |
| 98 |  | 1 |  | 1 | 100.0 |  | 1 |  |  | 0 | 0.0 |
| 99 |  | 1 |  | 1 | 100.0 |  | 1 |  |  | 0 | 0.0 |
| 100 |  | 1 |  | 1 | 100.0 |  | 1 |  |  | 0 | 0.0 |
| 101 |  | 3 | 1 | 2 | 66.7 |  | 2 | 1 |  | 1 | 33.3 |
| 113 |  | 1 |  | 1 | 100.0 |  | 1 |  |  | 0 | 0.0 |
| **Total** |  | **219** | **6** | **213** | **97.3** |  | **52** | **59** | **108** | **167** | **76.3** |

**5. S Table 2. N-Specific antibody testing results among participants with a PCR-confirmed infection, by serology assay type by year (n=219)**

|  |  |  |  | **Year 2022 (Jun 8-20)** | |  |  |  | **Year 2021 (Jun 8-21; Jul 12-16)** | | |  |  | **Year 2020 (Jul 6-21; Aug 17-21)** | | | |  | | |  |
| --- | --- | --- | --- | --- | --- | --- | --- | --- | --- | --- | --- | --- | --- | --- | --- | --- | --- | --- | --- | --- | --- |
|  | **PCR-confirmed infection** | |  | **Roche Elecsys Anti-SARS-CoV-2** | | **Abbott Alinity SARS-CoV-2 IgG** | |  | **Roche Elecsys Anti-SARS-CoV-2** | | **Abbott Alinity SARS-CoV-2 IgG** | |  | **Roche Elecsys Anti-SARS-CoV-2** | | **Abbott Alinity SARS-CoV-2 IgG** | | | |  |  |
|  | **(first time)** | **(second time)** |  | **Cut-off index (COI)** | **Results** | **Sample to calibrators (S/C)** | **Results^a^** |  | **Cut-off index (COI)** | **Results** | **Sample to calibrators (S/C)** | **Results^a^** |  | **Cut-off index (COI)** | **Results** | | **Sample to calibrators (S/C)** | | **Results^a^** | | |
| **Case 1** | 2020/4/19 | -- |  | 2.37 | positive | 0.05 | negative |  | 6.88 | positive | 0.10 | negative |  | 17.40 | positive | 1.39 | | grayzone | | |  |
| **Case 2** | 2020/7/3 | -- |  | 7.57 | positive | 0.18 | negative |  | 13.80 | positive | 0.18 | negative |  | 60.90 | positive | 5.96 | | positive | | |  |
| **Case 3** | 2020/7/4 | -- |  | 11.10 | positive | 0.81 | grayzone |  | 0.09 | negative | 0.54 | grayzone |  | 0.07 | negative | 0.77 | | grayzone | | |  |
| **Case 4** | 2020/7/5 | -- |  | 0.28 | negative | 0.02 | negative |  | 0.52 | negative | 0.06 | negative |  | 1.86 | positive | 0.92 | | grayzone | | |  |
| **Case 5** | 2020/7/19 | 2020/9/11 |  | 1.63 | positive | 0.06 | negative |  | 8.20 | positive | 0.21 | negative |  | 0.08 | negative | 0.09 | | negative | | |  |
| **Case 6** | 2020/7/22 | -- |  | 5.44 | positive | 0.17 | negative |  | 18.70 | positive | 0.31 | negative |  | 0.08 | negative | 0.10 | | negative | | |  |
| **Case 7** | 2020/7/24 | -- |  | 35.00 | positive | 0.38 | negative |  | 61.70 | positive | 1.05 | grayzone |  | 0.08 | negative | 0.52 | | grayzone | | |  |
| **Case 8** | 2020/8/7 | -- |  | 20.70 | positive | 0.29 | negative |  | 24.50 | positive | 0.58 | grayzone |  | 0.07 | negative | 0.02 | | negative | | |  |
| **Case 9** | 2020/8/19 | -- |  | 3.95 | positive | 0.25 | negative |  | 13.60 | positive | 0.15 | negative |  | 0.07 | negative | 0.04 | | negative | | |  |
| **Case 10** | 2020/8/19 | -- |  | 32.70 | positive | 0.49 | negative |  | 0.57 | negative | 0.10 | negative |  | 0.08 | negative | 0.04 | | negative | | |  |
| **Case 11** | 2020/9/22 | -- |  | 10.60 | positive | 0.22 | negative |  | 19.20 | positive | 0.42 | negative |  | 0.08 | negative | 0.37 | | negative | | |  |
| **Case 12** | 2020/10/4 | -- |  | 1.14 | positive | 0.04 | negative |  | 6.98 | positive | 0.09 | negative |  | 0.08 | negative | -- | | -- | | |  |
| **Case 13** | 2020/10/30 | -- |  | 2.70 | positive | 0.24 | negative |  | 6.12 | positive | 0.17 | negative |  | 0.08 | negative | 0.09 | | negative | | |  |
| **Case 14** | 2020/11/26 | -- |  | 183.00 | positive | 2.38 | positive |  | 27.90 | positive | 0.80 | grayzone |  | 0.08 | negative | 0.09 | | negative | | |  |
| **Case 15** | 2020/12/30 | -- |  | 2.19 | positive | 0.06 | negative |  | 25.80 | positive | 0.42 | negative |  | 0.07 | negative | 0.07 | | negative | | |  |
| **Case 16** | 2021/1/2 | -- |  | 31.00 | positive | 0.38 | negative |  | 293.00 | positive | 4.42 | positive |  | 0.08 | negative | 0.02 | | negative | | |  |
| **Case 17** | 2021/1/2 | -- |  | 9.76 | positive | 0.39 | negative |  | 139.00 | positive | 1.72 | positive |  | 0.08 | negative | 0.09 | | negative | | |  |
| **Case 18** | 2021/1/5 | -- |  | 2.71 | positive | 0.07 | negative |  | 71.60 | positive | 0.45 | negative |  | 0.14 | negative | 0.03 | | negative | | |  |
| **Case 19** | 2021/1/6 | -- |  | 0.89 | negative | 0.04 | negative |  | 13.20 | positive | 0.37 | negative |  | 0.08 | negative | 0.02 | | negative | | |  |
| **Case 20** | 2021/1/20 | -- |  | 5.77 | positive | 0.05 | negative |  | 36.00 | positive | 0.59 | grayzone |  | 0.07 | negative | 0.02 | | negative | | |  |
| **Case 21** | 2021/2/3 | -- |  | 6.82 | positive | 0.19 | negative |  | 16.40 | positive | 0.68 | grayzone |  | 0.08 | negative | 0.02 | | negative | | |  |
| **Case 22** | 2021/4/26 | -- |  | 9.40 | positive | 0.35 | negative |  | 194.00 | positive | 3.40 | positive |  | 0.07 | negative | 0.03 | | negative | | |  |
| **Case 23** | 2021/5/9 | -- |  | 43.20 | positive | 0.82 | grayzone |  | 97.10 | positive | 7.01 | positive |  | 0.08 | negative | 0.05 | | negative | | |  |
| **Case 24** | 2021/7/4 | -- |  | 91.20 | positive | 1.87 | positive |  | 0.09 | negative | 0.02 | negative |  | 0.08 | negative | 0.02 | | negative | | |  |
| **Case 25** | 2021/7/19 | -- |  | 4.32 | positive | 0.15 | negative |  | 0.09 | negative | 0.01 | negative |  | 0.08 | negative | 0.02 | | negative | | |  |
| **Case 26** | 2021/7/20 | -- |  | 5.61 | positive | 0.07 | negative |  | 0.09 | negative | 0.01 | negative |  | 0.08 | negative | 0.01 | | negative | | |  |
| **Case 27** | 2021/7/23 | -- |  | 2.46 | positive | 0.10 | negative |  | 0.11 | negative | 0.05 | negative |  | 0.08 | negative | 0.04 | | negative | | |  |
| **Case 28** | 2021/8/2 | -- |  | 8.14 | positive | 0.24 | negative |  | 0.09 | negative | 0.04 | negative |  | 0.08 | negative | 0.10 | | negative | | |  |
| **Case 29** | 2021/8/3 | -- |  | 10.30 | positive | 0.39 | negative |  | 0.08 | negative | 0.04 | negative |  | 0.08 | negative | 0.06 | | negative | | |  |
| **Case 30** | 2021/8/5 | -- |  | 137.00 | positive | 1.92 | positive |  | 0.08 | negative | 0.05 | negative |  | 0.08 | negative | 0.02 | | negative | | |  |
| **Case 31** | 2021/8/6 | -- |  | 178.00 | positive | 4.29 | positive |  | 0.09 | negative | 0.03 | negative |  | 0.08 | negative | 0.03 | | negative | | |  |
| **Case 32** | 2021/8/9 | -- |  | 28.30 | positive | 0.33 | negative |  | 0.09 | negative | 0.01 | negative |  | 0.08 | negative | 0.01 | | negative | | |  |
| **Case 33** | 2021/8/12 | -- |  | 4.31 | positive | 4.64 | positive |  | 0.08 | negative | 0.02 | negative |  | 0.08 | negative | 0.02 | | negative | | |  |
| **Case 34** | 2021/8/14 | -- |  | 21.00 | positive | 0.51 | grayzone |  | 0.09 | negative | 0.08 | negative |  | 0.07 | negative | 0.12 | | negative | | |  |
| **Case 35** | 2021/8/15 | -- |  | 105.00 | positive | 1.12 | grayzone |  | 0.09 | negative | 0.08 | negative |  | 0.08 | negative | 0.02 | | negative | | |  |
| **Case 36** | 2021/8/15 | -- |  | 206.00 | positive | 4.45 | positive |  | 0.09 | negative | 0.02 | negative |  | 0.08 | negative | 0.02 | | negative | | |  |
| **Case 37** | 2021/8/18 | -- |  | 181.00 | positive | 2.90 | positive |  | 0.09 | negative | 0.04 | negative |  | 0.08 | negative | 0.04 | | negative | | |  |
| **Case 38** | 2021/8/19 | -- |  | 91.90 | positive | 1.21 | grayzone |  | 0.09 | negative | 0.02 | negative |  | 0.08 | negative | 0.02 | | negative | | |  |
| **Case 39** | 2021/8/20 | -- |  | 21.30 | positive | 0.38 | negative |  | 0.09 | negative | 0.02 | negative |  | 0.08 | negative | 0.02 | | negative | | |  |
| **Case 40** | 2021/8/25 | -- |  | 17.30 | positive | 0.51 | grayzone |  | 0.09 | negative | 0.05 | negative |  | 0.08 | negative | 0.16 | | negative | | |  |
| **Case 41** | 2021/8/30 | -- |  | 19.50 | positive | 0.82 | grayzone |  | 0.09 | negative | 0.06 | negative |  | 0.08 | negative | 0.10 | | negative | | |  |
| **Case 42** | 2021/9/4 | -- |  | 16.40 | positive | 0.29 | negative |  | 0.08 | negative | 0.02 | negative |  | 0.07 | negative | 0.02 | | negative | | |  |
| **Case 43** | 2021/9/5 | -- |  | 12.80 | positive | 0.19 | negative |  | 0.09 | negative | 0.01 | negative |  | 0.08 | negative | 0.01 | | negative | | |  |
| **Case 44** | 2021/9/7 | -- |  | 1.31 | positive | 0.14 | negative |  | 0.09 | negative | 0.02 | negative |  | 0.07 | negative | 0.03 | | negative | | |  |
| **Case 45** | 2021/9/8 | -- |  | 178.00 | positive | 5.06 | positive |  | 0.10 | negative | 0.24 | negative |  | 0.08 | negative | 0.13 | | negative | | |  |
| **Case 46** | 2021/9/13 | -- |  | 116.00 | positive | 1.14 | grayzone |  | 0.08 | negative | 0.10 | negative |  | 0.07 | negative | 0.03 | | negative | | |  |
| **Case 47** | 2021/9/13 | -- |  | 5.46 | positive | 0.13 | negative |  | 0.10 | negative | 0.05 | negative |  | 0.08 | negative | 0.01 | | negative | | |  |
| **Case 48** | 2021/9/17 | -- |  | 146.00 | positive | 3.64 | positive |  | 0.10 | negative | 0.07 | negative |  | 0.07 | negative | 0.03 | | negative | | |  |
| **Case 49** | 2021/9/20 | -- |  | 9.42 | positive | 0.42 | negative |  | 0.09 | negative | 0.02 | negative |  | 0.07 | negative | 0.02 | | negative | | |  |
| **Case 50** | 2022/1/9 | -- |  | 1.17 | positive | 1.18 | grayzone |  | 0.09 | negative | 0.37 | negative |  | 0.08 | negative | 0.24 | | negative | | |  |
| **Case 51** | 2022/1/10 | -- |  | 2.22 | positive | 0.17 | negative |  | 0.10 | negative | 0.02 | negative |  | 0.08 | negative | 0.02 | | negative | | |  |
| **Case 52** | 2022/1/11 | -- |  | 0.06 | negative | 0.86 | grayzone |  | 0.09 | negative | 0.04 | negative |  | 0.08 | negative | 0.08 | | negative | | |  |
| **Case 53** | 2022/1/16 | -- |  | 8.66 | positive | 0.34 | negative |  | 0.08 | negative | 0.04 | negative |  | 0.07 | negative | 0.17 | | negative | | |  |
| **Case 54** | 2022/1/20 | -- |  | 36.40 | positive | 0.94 | grayzone |  | 0.09 | negative | 0.10 | negative |  | 0.07 | negative | 0.18 | | negative | | |  |
| **Case 55** | 2022/1/23 | -- |  | 18.70 | positive | 0.72 | grayzone |  | 0.09 | negative | 0.02 | negative |  | 0.08 | negative | 0.03 | | negative | | |  |
| **Case 56** | 2022/1/25 | -- |  | 4.76 | positive | 0.75 | grayzone |  | 0.09 | negative | 0.25 | negative |  | 0.08 | negative | 0.85 | | grayzone | | |  |
| **Case 57** | 2022/1/25 | -- |  | 37.70 | positive | 1.56 | positive |  | 0.09 | negative | 0.03 | negative |  | 0.08 | negative | 0.07 | | negative | | |  |
| **Case 58** | 2022/1/26 | -- |  | 15.90 | positive | 0.31 | negative |  | 0.09 | negative | 0.04 | negative |  | 0.08 | negative | 0.03 | | negative | | |  |
| **Case 59** | 2022/1/27 | -- |  | 20.70 | positive | 0.69 | grayzone |  | 0.10 | negative | 0.02 | negative |  | 0.07 | negative | 0.05 | | negative | | |  |
| **Case 60** | 2022/1/27 | -- |  | 7.44 | positive | 0.47 | negative |  | 0.09 | negative | 0.08 | negative |  | 0.08 | negative | 0.07 | | negative | | |  |
| **Case 61** | 2022/1/28 | -- |  | 48.00 | positive | 1.25 | grayzone |  | 0.09 | negative | 0.04 | negative |  | 0.07 | negative | 0.03 | | negative | | |  |
| **Case 62** | 2022/1/28 | -- |  | 10.10 | positive | 0.50 | grayzone |  | 0.09 | negative | 0.12 | negative |  | 0.07 | negative | 0.07 | | negative | | |  |
| **Case 63** | 2022/1/29 | -- |  | 57.20 | positive | 1.04 | grayzone |  | 0.09 | negative | 0.04 | negative |  | 0.08 | negative | -- | | -- | | |  |
| **Case 64** | 2022/1/29 | -- |  | 69.10 | positive | 1.98 | positive |  | 0.09 | negative | 0.19 | negative |  | 0.08 | negative | 0.07 | | negative | | |  |
| **Case 65** | 2022/1/30 | -- |  | 9.33 | positive | 0.63 | grayzone |  | 0.09 | negative | 0.10 | negative |  | 0.07 | negative | 0.04 | | negative | | |  |
| **Case 66** | 2022/1/30 | -- |  | 4.07 | positive | 0.19 | negative |  | 0.09 | negative | 0.02 | negative |  | 0.08 | negative | 0.01 | | negative | | |  |
| **Case 67** | 2022/1/31 | -- |  | 23.80 | positive | 1.13 | grayzone |  | 0.10 | negative | 0.01 | negative |  | 0.08 | negative | 0.03 | | negative | | |  |
| **Case 68** | 2022/2/1 | -- |  | 10.40 | positive | 0.95 | grayzone |  | 0.08 | negative | 0.20 | negative |  | 0.08 | negative | 0.06 | | negative | | |  |
| **Case 69** | 2022/2/1 | -- |  | 7.22 | positive | 1.14 | grayzone |  | 0.09 | negative | 0.68 | grayzone |  | 0.07 | negative | 0.51 | | grayzone | | |  |
| **Case 70** | 2022/2/3 | -- |  | 5.93 | positive | 0.29 | negative |  | 0.08 | negative | 0.02 | negative |  | 0.08 | negative | 0.02 | | negative | | |  |
| **Case 71** | 2022/2/3 | -- |  | 60.00 | positive | 1.04 | grayzone |  | 0.09 | negative | 0.03 | negative |  | 0.08 | negative | -- | | -- | | |  |
| **Case 72** | 2022/2/3 | -- |  | 19.50 | positive | 1.85 | positive |  | 0.08 | negative | 0.02 | negative |  | 0.08 | negative | 0.02 | | negative | | |  |
| **Case 73** | 2022/2/4 | -- |  | 7.56 | positive | 0.60 | grayzone |  | 0.09 | negative | 0.01 | negative |  | 0.08 | negative | 0.01 | | negative | | |  |
| **Case 74** | 2022/2/4 | -- |  | 10.80 | positive | 0.46 | negative |  | 0.09 | negative | 0.03 | negative |  | 0.08 | negative | 0.05 | | negative | | |  |
| **Case 75** | 2022/2/5 | -- |  | 11.70 | positive | 0.30 | negative |  | 0.09 | negative | 0.01 | negative |  | 0.08 | negative | 0.01 | | negative | | |  |
| **Case 76** | 2022/2/5 | -- |  | 11.90 | positive | 0.46 | negative |  | 0.09 | negative | 0.14 | negative |  | 0.07 | negative | 0.13 | | negative | | |  |
| **Case 77** | 2022/2/6 | -- |  | 2.80 | positive | 1.18 | grayzone |  | 0.09 | negative | 0.02 | negative |  | 0.07 | negative | 0.05 | | negative | | |  |
| **Case 78** | 2022/2/6 | -- |  | 87.90 | positive | 4.46 | positive |  | 0.10 | negative | 0.04 | negative |  | 0.08 | negative | 0.04 | | negative | | |  |
| **Case 79** | 2022/2/7 | -- |  | 39.40 | positive | 0.76 | grayzone |  | 0.35 | negative | 0.02 | negative |  | 0.23 | negative | 0.03 | | negative | | |  |
| **Case 80** | 2022/2/7 | -- |  | 56.50 | positive | 2.24 | positive |  | 0.08 | negative | 0.18 | negative |  | 0.08 | negative | 0.35 | | negative | | |  |
| **Case 81** | 2022/2/8 | -- |  | 19.80 | positive | 1.44 | positive |  | 0.09 | negative | 0.46 | negative |  | 0.08 | negative | 0.20 | | negative | | |  |
| **Case 82** | 2022/2/8 | -- |  | 79.00 | positive | 1.91 | positive |  | 0.09 | negative | 0.05 | negative |  | 0.08 | negative | 0.03 | | negative | | |  |
| **Case 83** | 2022/2/10 | -- |  | 5.35 | positive | 0.40 | negative |  | 0.10 | negative | 0.02 | negative |  | 0.08 | negative | 0.04 | | negative | | |  |
| **Case 84** | 2022/2/10 | -- |  | 15.30 | positive | 0.48 | negative |  | 0.10 | negative | 0.10 | negative |  | 0.08 | negative | 0.03 | | negative | | |  |
| **Case 85** | 2022/2/12 | -- |  | 13.00 | positive | 0.43 | negative |  | 0.09 | negative | 0.06 | negative |  | 0.07 | negative | 0.11 | | negative | | |  |
| **Case 86** | 2022/2/13 | -- |  | 20.20 | positive | 1.83 | positive |  | 0.09 | negative | 0.09 | negative |  | 0.08 | negative | 0.61 | | grayzone | | |  |
| **Case 87** | 2022/2/13 | -- |  | 2.61 | positive | 0.31 | negative |  | 0.09 | negative | 0.02 | negative |  | 0.07 | negative | 0.02 | | negative | | |  |
| **Case 88** | 2022/2/13 | -- |  | 7.97 | positive | 0.57 | grayzone |  | 0.09 | negative | 0.03 | negative |  | 0.08 | negative | 0.02 | | negative | | |  |
| **Case 89** | 2022/2/14 | -- |  | 8.29 | positive | 1.04 | grayzone |  | 0.09 | negative | 0.02 | negative |  | 0.08 | negative | 0.03 | | negative | | |  |
| **Case 90** | 2022/2/14 | -- |  | 8.80 | positive | 0.58 | grayzone |  | 0.09 | negative | 0.02 | negative |  | 0.07 | negative | 0.02 | | negative | | |  |
| **Case 91** | 2022/2/15 | -- |  | 48.50 | positive | 1.07 | grayzone |  | 0.09 | negative | 0.16 | negative |  | 0.08 | negative | 0.22 | | negative | | |  |
| **Case 92** | 2022/2/15 | -- |  | 14.10 | positive | 0.59 | grayzone |  | 0.09 | negative | 0.01 | negative |  | 0.08 | negative | 0.01 | | negative | | |  |
| **Case 93** | 2022/2/16 | -- |  | 4.32 | positive | 0.85 | grayzone |  | 0.10 | negative | 0.03 | negative |  | 0.07 | negative | 0.03 | | negative | | |  |
| **Case 94** | 2022/2/16 | -- |  | 19.30 | positive | 1.98 | positive |  | 0.09 | negative | 0.04 | negative |  | 0.08 | negative | 0.08 | | negative | | |  |
| **Case 95** | 2022/2/16 | -- |  | 3.20 | positive | 1.40 | positive |  | 0.09 | negative | 0.08 | negative |  | 0.08 | negative | 0.09 | | negative | | |  |
| **Case 96** | 2022/2/17 | -- |  | 14.90 | positive | 0.48 | negative |  | 0.09 | negative | 0.02 | negative |  | 0.07 | negative | 0.02 | | negative | | |  |
| **Case 97** | 2022/2/17 | -- |  | 71.90 | positive | 1.76 | positive |  | 0.09 | negative | 0.07 | negative |  | 0.08 | negative | 0.08 | | negative | | |  |
| **Case 98** | 2022/2/17 | -- |  | 38.30 | positive | 1.13 | grayzone |  | 0.09 | negative | 0.05 | negative |  | 0.08 | negative | 0.03 | | negative | | |  |
| **Case 99** | 2022/2/17 | -- |  | 36.30 | positive | 1.51 | positive |  | 0.08 | negative | 0.03 | negative |  | 0.08 | negative | 0.02 | | negative | | |  |
| **Case 100** | 2022/2/18 | -- |  | 0.06 | negative | 0.03 | negative |  | 0.09 | negative | 0.04 | negative |  | 0.08 | negative | 0.06 | | negative | | |  |
| **Case 101** | 2022/2/19 | -- |  | 53.60 | positive | 1.57 | positive |  | 0.09 | negative | 0.30 | negative |  | 0.08 | negative | 0.15 | | negative | | |  |
| **Case 102** | 2022/2/20 | -- |  | 5.32 | positive | 0.32 | negative |  | 0.09 | negative | 0.04 | negative |  | 0.08 | negative | 0.07 | | negative | | |  |
| **Case 103** | 2022/2/21 | -- |  | 36.80 | positive | 1.62 | positive |  | 0.10 | negative | 0.04 | negative |  | 0.08 | negative | 0.04 | | negative | | |  |
| **Case 104** | 2022/2/21 | -- |  | 25.90 | positive | 1.47 | positive |  | 0.09 | negative | 0.09 | negative |  | 0.07 | negative | 0.17 | | negative | | |  |
| **Case 105** | 2022/2/21 | -- |  | 37.60 | positive | 0.95 | grayzone |  | 0.09 | negative | 0.31 | negative |  | 0.07 | negative | 0.16 | | negative | | |  |
| **Case 106** | 2022/2/22 | -- |  | 7.07 | positive | 0.29 | negative |  | 0.09 | negative | 0.01 | negative |  | 0.07 | negative | 0.01 | | negative | | |  |
| **Case 107** | 2022/2/23 | -- |  | 21.80 | positive | 1.94 | positive |  | 0.09 | negative | 0.11 | negative |  | 0.08 | negative | 0.13 | | negative | | |  |
| **Case 108** | 2022/2/26 | -- |  | 53.90 | positive | 2.18 | positive |  | 0.75 | negative | 0.07 | negative |  | 0.24 | negative | 0.24 | | negative | | |  |
| **Case 109** | 2022/2/27 | -- |  | 7.98 | positive | 1.10 | grayzone |  | 0.09 | negative | 0.23 | negative |  | 0.08 | negative | 0.79 | | grayzone | | |  |
| **Case 110** | 2022/2/28 | -- |  | 32.10 | positive | 1.35 | grayzone |  | 0.09 | negative | 0.15 | negative |  | 0.08 | negative | 0.11 | | negative | | |  |
| **Case 111** | 2022/2/28 | -- |  | 31.10 | positive | 1.14 | grayzone |  | 0.10 | negative | 0.31 | negative |  | 0.07 | negative | 0.14 | | negative | | |  |
| **Case 112** | 2022/2/28 | -- |  | 14.30 | positive | 1.65 | positive |  | 0.09 | negative | 0.46 | negative |  | 0.08 | negative | 0.44 | | negative | | |  |
| **Case 113** | 2022/2/28 | -- |  | 7.71 | positive | 0.79 | grayzone |  | 0.08 | negative | 0.07 | negative |  | 0.07 | negative | 0.03 | | negative | | |  |
| **Case 114** | 2022/3/1 | -- |  | 6.21 | positive | 0.57 | grayzone |  | 0.09 | negative | 0.04 | negative |  | 0.08 | negative | 0.07 | | negative | | |  |
| **Case 115** | 2022/3/1 | -- |  | 27.30 | positive | 1.21 | grayzone |  | 0.09 | negative | 0.03 | negative |  | 0.08 | negative | 0.02 | | negative | | |  |
| **Case 116** | 2022/3/4 | -- |  | 3.29 | positive | 0.50 | grayzone |  | 0.09 | negative | 0.04 | negative |  | 0.07 | negative | 0.03 | | negative | | |  |
| **Case 117** | 2022/3/4 | -- |  | 6.59 | positive | 0.80 | grayzone |  | 0.08 | negative | 0.04 | negative |  | 0.08 | negative | 0.04 | | negative | | |  |
| **Case 118** | 2022/3/6 | -- |  | 24.60 | positive | 1.78 | positive |  | 0.09 | negative | 0.04 | negative |  | 0.08 | negative | 0.03 | | negative | | |  |
| **Case 119** | 2022/3/9 | -- |  | 75.40 | positive | 1.70 | positive |  | 0.10 | negative | 0.06 | negative |  | 0.08 | negative | 0.07 | | negative | | |  |
| **Case 120** | 2022/3/11 | -- |  | 21.70 | positive | 2.84 | positive |  | 0.09 | negative | 0.12 | negative |  | 0.08 | negative | 0.14 | | negative | | |  |
| **Case 121** | 2022/3/11 | -- |  | 10.80 | positive | 1.52 | positive |  | 0.09 | negative | 0.03 | negative |  | 0.08 | negative | 0.06 | | negative | | |  |
| **Case 122** | 2022/3/15 | -- |  | 5.62 | positive | 1.37 | grayzone |  | 0.09 | negative | 0.28 | negative |  | 0.08 | negative | 0.33 | | negative | | |  |
| **Case 123** | 2022/3/16 | -- |  | 22.90 | positive | 1.29 | grayzone |  | 0.09 | negative | 0.10 | negative |  | 0.07 | negative | 0.13 | | negative | | |  |
| **Case 124** | 2022/3/17 | -- |  | 7.85 | positive | 0.95 | grayzone |  | 0.09 | negative | 0.04 | negative |  | 0.08 | negative | 0.15 | | negative | | |  |
| **Case 125** | 2022/3/17 | -- |  | 9.41 | positive | 0.74 | grayzone |  | 0.09 | negative | 0.16 | negative |  | 0.08 | negative | 0.06 | | negative | | |  |
| **Case 126** | 2022/3/20 | -- |  | 108.00 | positive | 3.90 | positive |  | 0.09 | negative | 0.01 | negative |  | 0.07 | negative | 0.01 | | negative | | |  |
| **Case 127** | 2022/3/20 | -- |  | 7.51 | positive | 0.76 | grayzone |  | 0.11 | negative | 0.05 | negative |  | 0.08 | negative | 0.04 | | negative | | |  |
| **Case 128** | 2022/3/21 | -- |  | 23.10 | positive | 1.90 | positive |  | 0.09 | negative | 0.06 | negative |  | 0.08 | negative | 0.12 | | negative | | |  |
| **Case 129** | 2022/3/21 | -- |  | 14.60 | positive | 1.84 | positive |  | 0.09 | negative | 0.05 | negative |  | 0.08 | negative | -- | | -- | | |  |
| **Case 130** | 2022/3/22 | -- |  | 48.60 | positive | 3.78 | positive |  | 0.08 | negative | 0.02 | negative |  | 0.08 | negative | 0.02 | | negative | | |  |
| **Case 131** | 2022/3/23 | -- |  | 35.90 | positive | 2.22 | positive |  | 0.09 | negative | 0.03 | negative |  | 0.07 | negative | 0.02 | | negative | | |  |
| **Case 132** | 2022/3/23 | -- |  | 0.05 | negative | 2.67 | positive |  | 0.10 | negative | 0.01 | negative |  | 0.08 | negative | 0.05 | | negative | | |  |
| **Case 133** | 2022/3/23 | -- |  | 49.50 | positive | 1.84 | positive |  | 0.09 | negative | 0.04 | negative |  | 0.08 | negative | 0.10 | | negative | | |  |
| **Case 134** | 2022/3/23 | -- |  | 60.30 | positive | 3.96 | positive |  | 0.09 | negative | 0.07 | negative |  | 0.08 | negative | 0.03 | | negative | | |  |
| **Case 135** | 2022/3/24 | -- |  | 33.90 | positive | 2.25 | positive |  | 0.09 | negative | 0.04 | negative |  | 0.08 | negative | 0.06 | | negative | | |  |
| **Case 136** | 2022/3/25 | -- |  | 108.00 | positive | 5.07 | positive |  | 0.09 | negative | 0.18 | negative |  | 0.08 | negative | 0.18 | | negative | | |  |
| **Case 137** | 2022/3/25 | -- |  | 6.38 | positive | 1.49 | positive |  | 0.10 | negative | 0.08 | negative |  | 0.07 | negative | -- | | -- | | |  |
| **Case 138** | 2022/3/25 | -- |  | 13.90 | positive | 1.26 | grayzone |  | 0.09 | negative | 0.02 | negative |  | 0.08 | negative | 0.02 | | negative | | |  |
| **Case 139** | 2022/3/25 | -- |  | 81.90 | positive | 4.15 | positive |  | 0.08 | negative | 0.09 | negative |  | 0.08 | negative | 0.35 | | negative | | |  |
| **Case 140** | 2022/3/26 | -- |  | 3.48 | positive | 1.02 | grayzone |  | 0.09 | negative | 0.02 | negative |  | 0.07 | negative | 0.03 | | negative | | |  |
| **Case 141** | 2022/3/26 | -- |  | 29.10 | positive | 1.64 | positive |  | 0.09 | negative | 0.14 | negative |  | 0.08 | negative | 0.22 | | negative | | |  |
| **Case 142** | 2022/3/26 | -- |  | 41.40 | positive | 2.47 | positive |  | 0.13 | negative | 0.01 | negative |  | 0.09 | negative | 0.02 | | negative | | |  |
| **Case 143** | 2022/3/27 | -- |  | 15.90 | positive | 1.35 | grayzone |  | 0.09 | negative | 0.01 | negative |  | 0.08 | negative | 0.04 | | negative | | |  |
| **Case 144** | 2022/3/27 | -- |  | 64.30 | positive | 2.51 | positive |  | 0.09 | negative | 0.01 | negative |  | 0.08 | negative | -- | | -- | | |  |
| **Case 145** | 2022/3/28 | -- |  | 19.70 | positive | 2.34 | positive |  | 0.09 | negative | 0.02 | negative |  | 0.07 | negative | 0.03 | | negative | | |  |
| **Case 146** | 2022/3/28 | -- |  | 20.40 | positive | 1.13 | grayzone |  | 0.09 | negative | 0.24 | negative |  | 0.07 | negative | 0.19 | | negative | | |  |
| **Case 147** | 2022/3/29 | -- |  | 10.60 | positive | 1.69 | positive |  | 0.09 | negative | 0.13 | negative |  | 0.07 | negative | 0.11 | | negative | | |  |
| **Case 148** | 2022/3/30 | -- |  | 66.50 | positive | 2.45 | positive |  | 0.10 | negative | 0.05 | negative |  | 0.08 | negative | 0.03 | | negative | | |  |
| **Case 149** | 2022/3/31 | -- |  | 4.44 | positive | 0.74 | grayzone |  | 0.09 | negative | 0.05 | negative |  | 0.08 | negative | 0.04 | | negative | | |  |
| **Case 150** | 2022/3/31 | -- |  | 93.00 | positive | 2.22 | positive |  | 0.09 | negative | 0.53 | grayzone |  | 0.08 | negative | 0.15 | | negative | | |  |
| **Case 151** | 2022/4/1 | -- |  | 13.30 | positive | 0.65 | grayzone |  | 0.09 | negative | 0.04 | negative |  | 0.08 | negative | -- | | -- | | |  |
| **Case 152** | 2022/4/1 | -- |  | 63.00 | positive | 3.41 | positive |  | 0.09 | negative | 0.12 | negative |  | 0.08 | negative | 0.06 | | negative | | |  |
| **Case 153** | 2022/4/1 | -- |  | 60.40 | positive | 4.52 | positive |  | 0.10 | negative | 0.22 | negative |  | 0.08 | negative | 0.13 | | negative | | |  |
| **Case 154** | 2022/4/2 | -- |  | 122.00 | positive | 4.39 | positive |  | 0.09 | negative | 0.17 | negative |  | 0.07 | negative | 0.10 | | negative | | |  |
| **Case 155** | 2022/4/2 | -- |  | 26.30 | positive | 2.44 | positive |  | 0.09 | negative | 0.06 | negative |  | 0.08 | negative | 0.04 | | negative | | |  |
| **Case 156** | 2022/4/2 | -- |  | 199.00 | positive | 4.79 | positive |  | 20.90 | positive | 0.37 | negative |  | 0.08 | negative | 0.01 | | negative | | |  |
| **Case 157** | 2022/4/2 | -- |  | 95.00 | positive | 5.70 | positive |  | 0.09 | negative | 0.10 | negative |  | 0.08 | negative | 0.12 | | negative | | |  |
| **Case 158** | 2022/4/2 | -- |  | 29.20 | positive | 1.62 | positive |  | 0.09 | negative | 0.08 | negative |  | 0.08 | negative | 0.08 | | negative | | |  |
| **Case 159** | 2022/4/2 | -- |  | 41.80 | positive | 2.47 | positive |  | 0.09 | negative | 0.02 | negative |  | 0.08 | negative | 0.02 | | negative | | |  |
| **Case 160** | 2022/4/2 | -- |  | 17.20 | positive | 1.11 | grayzone |  | 0.09 | negative | 0.01 | negative |  | 0.08 | negative | 0.01 | | negative | | |  |
| **Case 161** | 2022/4/2 | -- |  | 22.10 | positive | 1.41 | positive |  | 0.08 | negative | 0.01 | negative |  | 0.08 | negative | 0.01 | | negative | | |  |
| **Case 162** | 2022/4/3 | -- |  | 14.00 | positive | 1.78 | positive |  | 0.09 | negative | 0.05 | negative |  | 0.08 | negative | 0.07 | | negative | | |  |
| **Case 163** | 2022/4/3 | -- |  | 2.80 | positive | 0.80 | grayzone |  | 0.08 | negative | 0.01 | negative |  | 0.07 | negative | 0.02 | | negative | | |  |
| **Case 164** | 2022/4/3 | -- |  | 109.00 | positive | 3.86 | positive |  | 0.09 | negative | 0.01 | negative |  | 0.08 | negative | 0.01 | | negative | | |  |
| **Case 165** | 2022/4/4 | -- |  | 152.00 | positive | 4.25 | positive |  | 11.30 | positive | 0.66 | grayzone |  | 0.07 | negative | 0.32 | | negative | | |  |
| **Case 166** | 2022/4/4 | -- |  | 16.90 | positive | 2.54 | positive |  | 0.09 | negative | 0.25 | negative |  | 0.08 | negative | 0.33 | | negative | | |  |
| **Case 167** | 2022/4/4 | -- |  | 40.40 | positive | 2.88 | positive |  | 0.42 | negative | 0.05 | negative |  | 0.30 | negative | 0.03 | | negative | | |  |
| **Case 168** | 2022/4/5 | -- |  | 18.70 | positive | 1.64 | positive |  | 0.10 | negative | 0.13 | negative |  | 0.08 | negative | 0.09 | | negative | | |  |
| **Case 169** | 2022/4/6 | -- |  | 32.00 | positive | 1.83 | positive |  | 0.08 | negative | 0.01 | negative |  | 0.07 | negative | 0.01 | | negative | | |  |
| **Case 170** | 2022/4/6 | -- |  | 36.40 | positive | 2.37 | positive |  | 0.09 | negative | 0.08 | negative |  | 0.07 | negative | 0.06 | | negative | | |  |
| **Case 171** | 2022/4/6 | -- |  | 46.50 | positive | 2.63 | positive |  | 0.10 | negative | 0.02 | negative |  | 0.07 | negative | 0.02 | | negative | | |  |
| **Case 172** | 2022/4/6 | -- |  | 36.00 | positive | 1.57 | positive |  | 0.10 | negative | 0.03 | negative |  | 0.08 | negative | 0.04 | | negative | | |  |
| **Case 173** | 2022/4/7 | -- |  | 31.20 | positive | 2.42 | positive |  | 0.09 | negative | 0.02 | negative |  | 0.07 | negative | -- | | -- | | |  |
| **Case 174** | 2022/4/7 | -- |  | 62.30 | positive | 3.09 | positive |  | 0.09 | negative | 0.03 | negative |  | 0.08 | negative | 0.04 | | negative | | |  |
| **Case 175** | 2022/4/8 | -- |  | 64.90 | positive | 3.65 | positive |  | 0.10 | negative | 0.08 | negative |  | 0.09 | negative | 0.13 | | negative | | |  |
| **Case 176** | 2022/4/10 | -- |  | 21.90 | positive | 1.99 | positive |  | 0.09 | negative | 0.03 | negative |  | 0.08 | negative | 0.04 | | negative | | |  |
| **Case 177** | 2022/4/10 | -- |  | 55.80 | positive | 2.43 | positive |  | 0.10 | negative | 0.09 | negative |  | 0.08 | negative | 0.08 | | negative | | |  |
| **Case 178** | 2022/4/12 | -- |  | 4.54 | positive | 0.82 | grayzone |  | 0.09 | negative | 0.41 | negative |  | 0.08 | negative | 0.26 | | negative | | |  |
| **Case 179** | 2022/4/12 | -- |  | 22.00 | positive | 2.25 | positive |  | 0.10 | negative | 0.03 | negative |  | 0.08 | negative | 0.06 | | negative | | |  |
| **Case 180** | 2022/4/16 | -- |  | 19.10 | positive | 1.68 | positive |  | 0.09 | negative | 0.02 | negative |  | 0.08 | negative | 0.02 | | negative | | |  |
| **Case 181** | 2022/4/18 | -- |  | 22.80 | positive | 3.61 | positive |  | 0.09 | negative | 0.05 | negative |  | 0.08 | negative | 0.08 | | negative | | |  |
| **Case 182** | 2022/4/19 | -- |  | 1.78 | positive | 0.47 | negative |  | 0.09 | negative | 0.02 | negative |  | 0.08 | negative | 0.02 | | negative | | |  |
| **Case 183** | 2022/4/20 | -- |  | 11.80 | positive | 2.05 | positive |  | 0.10 | negative | 0.11 | negative |  | 0.09 | negative | 0.09 | | negative | | |  |
| **Case 184** | 2022/4/20 | -- |  | 66.70 | positive | 0.75 | grayzone |  | 0.09 | negative | 0.01 | negative |  | 0.08 | negative | 0.01 | | negative | | |  |
| **Case 185** | 2022/4/21 | -- |  | 15.60 | positive | 2.56 | positive |  | 0.09 | negative | 0.05 | negative |  | 0.08 | negative | 0.07 | | negative | | |  |
| **Case 186** | 2022/4/22 | -- |  | 5.06 | positive | 0.88 | grayzone |  | 0.09 | negative | 0.01 | negative |  | 0.07 | negative | 0.01 | | negative | | |  |
| **Case 187** | 2022/4/23 | -- |  | 69.90 | positive | 3.68 | positive |  | 0.10 | negative | 0.21 | negative |  | 0.08 | negative | -- | | -- | | |  |
| **Case 188** | 2022/4/23 | -- |  | 91.70 | positive | 4.13 | positive |  | 0.09 | negative | 0.02 | negative |  | 0.08 | negative | 0.02 | | negative | | |  |
| **Case 189** | 2022/4/26 | -- |  | 17.50 | positive | 2.29 | positive |  | 0.09 | negative | 0.01 | negative |  | 0.08 | negative | 0.01 | | negative | | |  |
| **Case 190** | 2022/4/26 | -- |  | 12.60 | positive | 1.53 | positive |  | 0.09 | negative | 0.01 | negative |  | 0.08 | negative | 0.01 | | negative | | |  |
| **Case 191** | 2022/4/27 | -- |  | 6.13 | positive | 1.68 | positive |  | 0.10 | negative | 0.01 | negative |  | 0.07 | negative | 0.01 | | negative | | |  |
| **Case 192** | 2022/4/29 | -- |  | 42.60 | positive | 2.67 | positive |  | 0.09 | negative | 0.03 | negative |  | 0.08 | negative | 0.02 | | negative | | |  |
| **Case 193** | 2022/4/29 | -- |  | 23.10 | positive | 4.55 | positive |  | 0.09 | negative | 0.05 | negative |  | 0.08 | negative | 0.03 | | negative | | |  |
| **Case 194** | 2022/4/30 | -- |  | 18.80 | positive | 2.39 | positive |  | 0.10 | negative | 0.22 | negative |  | 0.08 | negative | 0.15 | | negative | | |  |
| **Case 195** | 2022/5/3 | -- |  | 46.80 | positive | 5.11 | positive |  | 0.09 | negative | 0.05 | negative |  | 0.08 | negative | 0.02 | | negative | | |  |
| **Case 196** | 2022/5/3 | -- |  | 15.20 | positive | 4.66 | positive |  | 0.09 | negative | 0.15 | negative |  | 0.07 | negative | 0.20 | | negative | | |  |
| **Case 197** | 2022/5/4 | -- |  | 42.20 | positive | 4.55 | positive |  | 0.08 | negative | 0.02 | negative |  | 0.07 | negative | 0.03 | | negative | | |  |
| **Case 198** | 2022/5/6 | -- |  | 30.30 | positive | 4.62 | positive |  | 0.11 | negative | 0.02 | negative |  | 0.08 | negative | 0.02 | | negative | | |  |
| **Case 199** | 2022/5/6 | -- |  | 51.00 | positive | 4.25 | positive |  | 0.10 | negative | 0.20 | negative |  | 0.08 | negative | 0.06 | | negative | | |  |
| **Case 200** | 2022/5/7 | -- |  | 157.00 | positive | 7.53 | positive |  | 0.09 | negative | 0.02 | negative |  | 0.08 | negative | 0.02 | | negative | | |  |
| **Case 201** | 2022/5/8 | -- |  | 2.28 | positive | 0.22 | negative |  | 0.28 | negative | 0.02 | negative |  | 0.08 | negative | 0.02 | | negative | | |  |
| **Case 202** | 2022/5/10 | -- |  | 14.80 | positive | 4.05 | positive |  | 0.09 | negative | 0.02 | negative |  | 0.08 | negative | 0.02 | | negative | | |  |
| **Case 203** | 2022/5/10 | -- |  | 6.18 | positive | 1.56 | positive |  | 0.09 | negative | 0.06 | negative |  | 0.08 | negative | 0.03 | | negative | | |  |
| **Case 204** | 2022/5/11 | -- |  | 26.40 | positive | 4.41 | positive |  | 0.09 | negative | 0.13 | negative |  | 0.07 | negative | 0.06 | | negative | | |  |
| **Case 205** | 2022/5/13 | -- |  | 4.07 | positive | 1.69 | positive |  | 0.10 | negative | 0.01 | negative |  | 0.08 | negative | 0.01 | | negative | | |  |
| **Case 206** | 2022/5/13 | -- |  | 8.89 | positive | 2.12 | positive |  | 0.09 | negative | 0.11 | negative |  | 0.08 | negative | 0.04 | | negative | | |  |
| **Case 207** | 2022/5/14 | -- |  | 82.60 | positive | 8.93 | positive |  | 6.12 | positive | 1.79 | positive |  | 0.07 | negative | 0.04 | | negative | | |  |
| **Case 208** | 2022/5/15 | -- |  | 0.06 | negative | 0.04 | negative |  | 0.09 | negative | 0.04 | negative |  | 0.08 | negative | 0.04 | | negative | | |  |
| **Case 209** | 2022/5/17 | -- |  | 6.04 | positive | 3.29 | positive |  | 0.08 | negative | 0.02 | negative |  | 0.07 | negative | 0.02 | | negative | | |  |
| **Case 210** | 2022/5/17 | -- |  | 44.40 | positive | 5.98 | positive |  | 0.09 | negative | 0.06 | negative |  | 0.08 | negative | 0.02 | | negative | | |  |
| **Case 211** | 2022/5/19 | -- |  | 1.64 | positive | 1.35 | grayzone |  | 0.09 | negative | 0.02 | negative |  | 0.08 | negative | 0.01 | | negative | | |  |
| **Case 212** | 2022/5/20 | -- |  | 16.60 | positive | 5.29 | positive |  | 0.09 | negative | 0.15 | negative |  | 0.07 | negative | 0.27 | | negative | | |  |
| **Case 213** | 2022/5/20 | -- |  | 9.87 | positive | 4.36 | positive |  | 0.09 | negative | 0.11 | negative |  | 0.08 | negative | 0.17 | | negative | | |  |
| **Case 214** | 2022/5/21 | -- |  | 9.48 | positive | 5.08 | positive |  | 0.09 | negative | 0.02 | negative |  | 0.07 | negative | 0.02 | | negative | | |  |
| **Case 215** | 2022/5/21 | -- |  | 1.46 | positive | 1.07 | grayzone |  | 0.09 | negative | 0.01 | negative |  | 0.08 | negative | 0.02 | | negative | | |  |
| **Case 216** | 2022/5/24 | -- |  | 23.80 | positive | 2.51 | positive |  | 0.22 | negative | 0.02 | negative |  | 0.18 | negative | 0.02 | | negative | | |  |
| **Case 217** | 2022/5/25 | -- |  | 10.00 | positive | 3.43 | positive |  | 0.09 | negative | 0.05 | negative |  | 0.08 | negative | 0.25 | | negative | | |  |
| **Case 218** | 2022/5/25 | -- |  | 17.50 | positive | 3.33 | positive |  | 0.09 | negative | 0.04 | negative |  | 0.08 | negative | 0.05 | | negative | | |  |
| **Case 219** | 2022/5/27 | -- |  | 18.30 | positive | 5.26 | positive |  | 0.10 | negative | 0.05 | negative |  | 0.08 | negative | 0.07 | | negative | | |  |

^a^ Testing results for Abbott Alinity SARS-CoV-2 IgG includes negative (<0.5), grayzone (0.5 to <1.4) and positive (≥1.4).
